# Supplementary material for: Erythropoietin directly remodels the clonal composition of murine hematopoietic multipotent progenitor cells
Source: eLife. 2022 Feb 15;11:e66922. doi: 10.7554/eLife.66922 (PMC8884727; doi:10.7554/eLife.66922)
Supplement: Supplementary file 1. — Same data as in Figure 1—figure supplement 4 and Figure 1—figure supplement 3. HSPCs were cultured with EPO (1000 ng/ml) for 16 hr. Barcodes in the erythroid (E), myeloid (M), B-lymphoid (B) lineage, dendritic cell (DC), and HSPCs were analyzed 4 weeks after transplantation and categorized by bias using a 10% threshold. The output of MB and ME classified barcodes to the B, M, and E lineages was analyzed using a permutation test. By permutating the mice of the control and EPO groups, the random distribution of this output was generated and compared to the real output difference between the control and EPO group. A p-value was generated by permutation testing. [file elife-66922-supp1.docx]

**Supplementary File 1: Permutation testing of changes in clonality after transplantation of EPO-exposed HSPCs.** Same data as in Figure 1 figure supplement 4 and Figure 1 figure supplement 3. HSPCs were cultured with EPO (1,000 ng/ml) for 16h. Barcodes in the erythroid (E), myeloid (M), B-lymphoid (B) lineage, dendritic cell (DC) and HSPCs, were analyzed four weeks after transplantation and categorized by bias using a 10% threshold. The output of MB and ME classified barcodes to the B, M, and E lineages was analyzed using a permutation test. By permutating the mice of control and EPO groups, the random distribution of this output was generated and compared to the real output difference between control and EPO group. A p-value was generated using permutation testing.

| **Figure 1 figure supplement** | **Condition** | **p-value** | | | |
| --- | --- | --- | --- | --- | --- |
|  |  | **MB in B** | **MB in M** | **ME in E** | **ME in M** |
| **3** | **HSPCs 1000 ng/ml repeat** | 0.01 | 0.0083 | 0.0087 | 0.0087 |
| **4** | **HSPCs 1000 ng/ml - 6 weeks timepoint** | 0.01 | 0.01 | 0.0138 | 0.0185 |
