# Supplementary material for: Erythropoietin directly remodels the clonal composition of murine hematopoietic multipotent progenitor cells
Source: eLife. 2022 Feb 15;11:e66922. doi: 10.7554/eLife.66922 (PMC8884727; doi:10.7554/eLife.66922)
Supplement: Transparent reporting form [file elife-66922-transrepform1.pdf]

eLIFE
Westbrook Centre, Milton Road
Cambridge CB4 1YG
UK
P 01223 855340
W elifesciences.org
T @elife

eLife's transparent reporting form

We encourage authors to provide detailed information *within their submission* to facilitate the interpretation and replication of experiments. Authors can upload supporting documentation to indicate the use of appropriate reporting guidelines for health-related research (see EQUATOR Network), life science research (see the BioSharing Information Resource), or the ARRIVE guidelines for reporting work involving animal research. Where applicable, authors should refer to any relevant reporting standards documents in this form.

If you have any questions, please consult our Journal Policies and/or contact us: editorial@elifesciences.org.

Sample-size estimation

- You should state whether an appropriate sample size was computed when the study was being designed
- You should state the statistical method of sample size computation and any required assumptions
- If no explicit power analysis was used, you should describe how you decided what sample (replicate) size (number) to use

Please outline where this information can be found within the submission (e.g., sections or figure legends), or explain why this information doesn't apply to your submission:

We performed preliminary experiments to assess effect size and could detect statistical differences with groups of three mice. Sample sizes are indicated in the figure and figure supplement legends.

Replicates

- You should report how often each experiment was performed
- You should include a definition of biological versus technical replication
- The data obtained should be provided and sufficient information should be provided to indicate the number of independent biological and/or technical replicates
- If you encountered any outliers, you should describe how these were handled
- Criteria for exclusion/inclusion of data should be clearly stated
- High-throughput sequence data should be uploaded before submission, with a private link for reviewers provided (these are available from both GEO and ArrayExpress)

Please outline where this information can be found within the submission (e.g., sections or figure legends), or explain why this information doesn't apply to your submission:

The main experimental cohort (4-week timepoint at EPO concentration 1000 ng/ml) was repeated twice with the exact same sorted and analyzed cell populations (Figure 1 and Figure 1 – figure supplement 3). Furthermore, the cohort was repeated with more detailed myeloid gating (Figure 3 and Figure 3 – figure supplement 1), with mature hematopoietic cell subsets sorted from spleen and bone (Figure 1 – figure supplement 2), and with HSPC sorted from bone (Figure 4). The experimental cohorts with different EPO concentrations (160 ng/ml, with and without additional EPO injection and 1000 ng/ml with additional injection of EPO) (Figure 2 and Figure 2- figure supplement 1), and with readout at different timepoints (6 weeks (Figure 1 – figure supplement 4) and 4 months (Figure 7)) were run once. Sample sizes for each experiment are indicated in the respective figure or figure supplement legend.

For each of the experimental cohorts, barcode analysis entailed the analysis of two technical replicates for every sample as in Naik et al 2013, DOI 10.1038/nature12013. More in detail, cell subsets collected through FACS were split into two fractions after cell lysis and processed independently throughout barcode amplification by PCR and sequencing. After sequencing, these technical replicates were used to remove erroneous barcodes based on correlation between them, and the mean of the technical replicates was further used for analysis. These steps are further detailed in the Material and Methods section.

The experimental cohorts were started with 4-5 mice per experimental condition (EPO vs control). The final sample sizes indicated in the respective figure or figure supplement legends result from 1) mice dying before readout 2) mice with an engraftment of donor cells of under 5% at read-out 3) filtering during barcode sequencing data analysis. More in detail, mice for which one or more cell subset samples did not pass the filtering steps as detailed in the Material and Methods section, could not be included in the analysis.

Statistical reporting

- Statistical analysis methods should be described and justified
- Raw data should be presented in figures whenever informative to do so (typically when N per group is less than 10)
- For each experiment, you should identify the statistical tests used, exact values of N, definitions of center, methods of multiple test correction, and dispersion and precision measures (e.g., mean, median, SD, SEM, confidence intervals; and, for the major substantive results, a measure of effect size (e.g., Pearson's r, Cohen's d)
- Report exact p-values wherever possible alongside the summary statistics and 95% confidence intervals. These should be reported for all key questions and not only when the p-value is less than 0.05.

Please outline where this information can be found within the submission (e.g., sections or figure legends), or explain why this information doesn't apply to your submission:

For each experiment, the statistical test used, exact value of N, and dispersion and precision measures are indicated in the figure or figure supplement legend. Statistics on barcoding results were performed using a permutation test as in Tak et al DOI https://doi.org/10.1101/586354, taking a p-value of 0.05 as threshold for significance. The p-values of the permutation tests are provided in Table 2 and Figure 1 – figure supplement 4. Data and code to perform the permutation test can be found at https://github.com/PerieTeam/Eisele-et-al.-. Significance of flow cytometry results was assessed using Student's T test taking a p-value of 0.05 as threshold for significance. For scRNAseq data analysis statistical testing was performed as indicated in the Material and Methods section and/or corresponding Figure and figure supplement legends.

(For large datasets, or papers with a very large number of statistical tests, you may upload a single table file with tests, Ns, etc., with reference to sections in the manuscript.)

Group allocation

- Indicate how samples were allocated into experimental groups (in the case of clinical studies, please specify allocation to treatment method); if randomization was used, please also state if restricted randomization was applied
- Indicate if masking was used during group allocation, data collection and/or data analysis

Please outline where this information can be found within the submission (e.g., sections or figure legends), or explain why this information doesn't apply to your submission:

Mice were allocated to groups randomly. No masking was used during group allocation, data collection and data analysis. This information can be found in the Material and Methods section.

Additional data files ("source data")

- We encourage you to upload relevant additional data files, such as numerical data that are represented as a graph in a figure, or as a summary table
- Where provided, these should be in the most useful format, and they can be uploaded as "Source data" files linked to a main figure or table
- Include model definition files including the full list of parameters used
- Include code used for data analysis (e.g., R, MatLab)
- Avoid stating that data files are "available upon request"

Please indicate the figures or tables for which source data files have been provided:

Data and code can be found at https://github.com/PerieTeam/Eisele-et-al.-. This link is also provided in the Material and Methods section.

eLife Sciences Publications, Ltd is a limited liability non- profit non-stock corporation incorporated in the State of Delaware,

USA, with company number 5030732, and is registered in the UK with company number FC030576 and2 branch number BR015634 at the address Westbrook Centre, Milton Road Cambridge CB4 1YG, UK | March 2019
